# Noninvasive 3D-head-scan used for 3D-printed customized helm by patient undergoing decompressive hemicraniectomy

**DOI:** 10.3389/fbioe.2025.1530126

**Published:** 2025-04-11

**Authors:** Fabian Kropla, Johannes Wach, Dirk Winkler, Ronny Grunert, Erdem Güresir, Martin Vychopen

**Affiliations:** ^1^ Department of Neurosurgery, University of Leipzig Medical Center, Leipzig, Germany; ^2^ Frauenhofer Institute for Machine Tools and Forming Technology, Zittau, Germany

**Keywords:** 3D printing, additive manufacturing, custom medical devices, neurosurgery, 3D imaging

## Abstract

**Introduction:**

Decompressive hemicraniectomy (DC) is a procedure used to treat elevated, therapy-refractory intracranial pressure. Despite the severity of the underlying pathology, selected patients quickly regain mobility and are at risk of secondary injury due to the post-craniotomy defect. A 3D-printed helmet offers a quickly available and safe solution. Up to now, postoperative CT scans have been used as a template for helmet construction. In this study, we present an alternative helmet construction using a non-invasive 3D scan (ArtecLeo, Artec3D), which is used to capture craniometrics data, plan the shape of the helmet, and compare it with routinely performed CT scans. A significant difference in defect displacement between supine scans and standing or sitting scans is evident, which is quantified.

**Methods:**

We included six patients who underwent decompressive craniectomy due to therapyrefractory elevation of intracranial pressure as a consequence of following pathologies: large intracerebral hemorrhage, large cerebral infarction, sever traumatic brain injury and poor grade subarachnoid hemorrhage. All patients underwent 3-D scan and subsequently, a helmet was created to cover the craniectomy area.

**Results:**

A surface heat-map comparison was performed to demonstrate the differences between the data obtained by 3-D scan (lying and sitting position) and CT-scan. Furthermore, the heat-map demonstrates the frontal and posterior surface difference between CT-scan and sitting position. Comparing the lying position 3-D scan and CT-scan, we were able to demonstrate a tissue shift, mainly in cranial and frontal areas.

**Conclusion:**

We demonstrated that non-invasive 3D-scan (Artec3D) is a feasible alternative to scan the head of the patients after DHC in order to construct a 3D-printed Helm. According to the heat-map analysis, it seems to be more reliable data assessment method in independently moving patients.

## Introduction

Decompressive hemicraniectomy (DC) is an emergent, life-saving procedure used in treatment of therapy refractory intracranial hypertension. According to the severity of underlying pathology, the mortality and morbidity of DC remains high (Ndiaye Sy et al., 2021). After initial recovery, the bone flap re-insertion (cranioplasty) needs to be performed to repair the cranial defect. According to contemporary literature, the complication rate of cranioplasty lowers, if this is performed at least 60 days after initial decompression (Corallo et al., 2014).

In such cases, the cerebral tissue remains exposed to atmospheric pressure, which might cause functional impairment by otherwise healthy brain (Schuss et al., 2012). Furthermore, in selected cases, good functional outcome with rapid neurological recovery is achieved. Such patients undergo intensive rehabilitation and are in need of helmet to prevent the potentially fatal injury (Kurland et al., 2015) of the non-protected cerebral tissue in case of fall. According to individual differences in neurocranium morphology (Noble et al., 2019), conventional pre-fabricated helmet might not be feasible for all patients (Schur et al., 2020).

3-D printed helmet has been recently presented as a quickly available, individualized and patient-tailored solution (Pang et al., 2022). However, in cases where patients are rapidly able to sit and walk independently, the assessment of the cranial defect in computer tomography (CT) might limit the adjustment as it does not optimally assess the transcalvarial hernia configuration (Liao et al., 2015) in upright position.

In our study, we propose a concept of 3-D printed helmet based on non-invasive 3-D head scan by ArtecLeo from Artec3D Performed by patients, who rapidly achieved mobility after decompressive 3D printed helmet based on data from an Artec 3D scan.

## Methods

We included six patients who underwent decompressive craniectomy due to therapyrefractory elevation of intracranial pressure as a consequence of following pathologies: large intracerebral hemorrhage, large cerebral infarction, sever traumatic brain injury and poor grade subarachnoid hemorrhage. All patients underwent rapid clinical recovery and were able to sit and walk independently. The 3D scan for the helmet was performed afterwards.

### Scanning

A CT scan (computed tomography) uses X-rays to create detailed cross-sectional images of the body’s internal structures, often used for medical diagnostics. In contrast, a 3D scan employs non-invasive techniques like structured light or laser technology to capture the external geometry of objects or body surfaces, focusing on surface detail rather than internal structures.

The most prevalent types of scanners are laser scanners and structured light scanners. Essentially, both scanner types operate in a similar manner by emitting light that is reflected by the object being captured (Haleem et al., 2022). Klicken oder tippen Sie hier, um Text einzugeben. This returning light is then captured by the receiving optics. Structured light scanners project light onto the object in a defined pattern. By analyzing this pattern, deformations and distortions of structures can be detected. The triangulation method is used to capture the spatial coordinates X, Y, and Z of individual measurement points (Javaid et al., 2021). In this process, angular measurements of individual triangles are carried out using trigonometric functions.

The number of acquired measurement points increases with longer scanning durations, resulting in a point cloud. Occasionally, multiple individual scans are generated during the scanning process, which are automatically merged and supplemented with texture through an optional camera system. An important parameter is the number of images captured per second.

For the investigation, the mobile handheld scanner ArtecLeo was utilized. This scanner offers the advantage of being deployable in operational environments and is remarkably user-friendly in terms of handling. The outcome is a surface model of the object, which can be edited and customized using the appropriate graphics software. Thanks to this high acquisition rate, it is possible to fully scan the head of the patient within a matter of minutes.

Subsequently, we compared the data obtained by ArtecLeo with the routine post-operative CT scan. The differences in a shape were presented in a form of a heat map created in software Zeiss Inspect (Carl Zeiss IQS Deutschland GmbH, 2024). The differences between a 3D scan, with the patient seated, and the CT scan, with the patient lying down, were presented in the form of a heat map generated using Zeiss Inspect software. This heat map provides a direct comparison of defect displacement, with the CT data used as the baseline actual data and the scan data as the target data.

**FIGURE 1:**
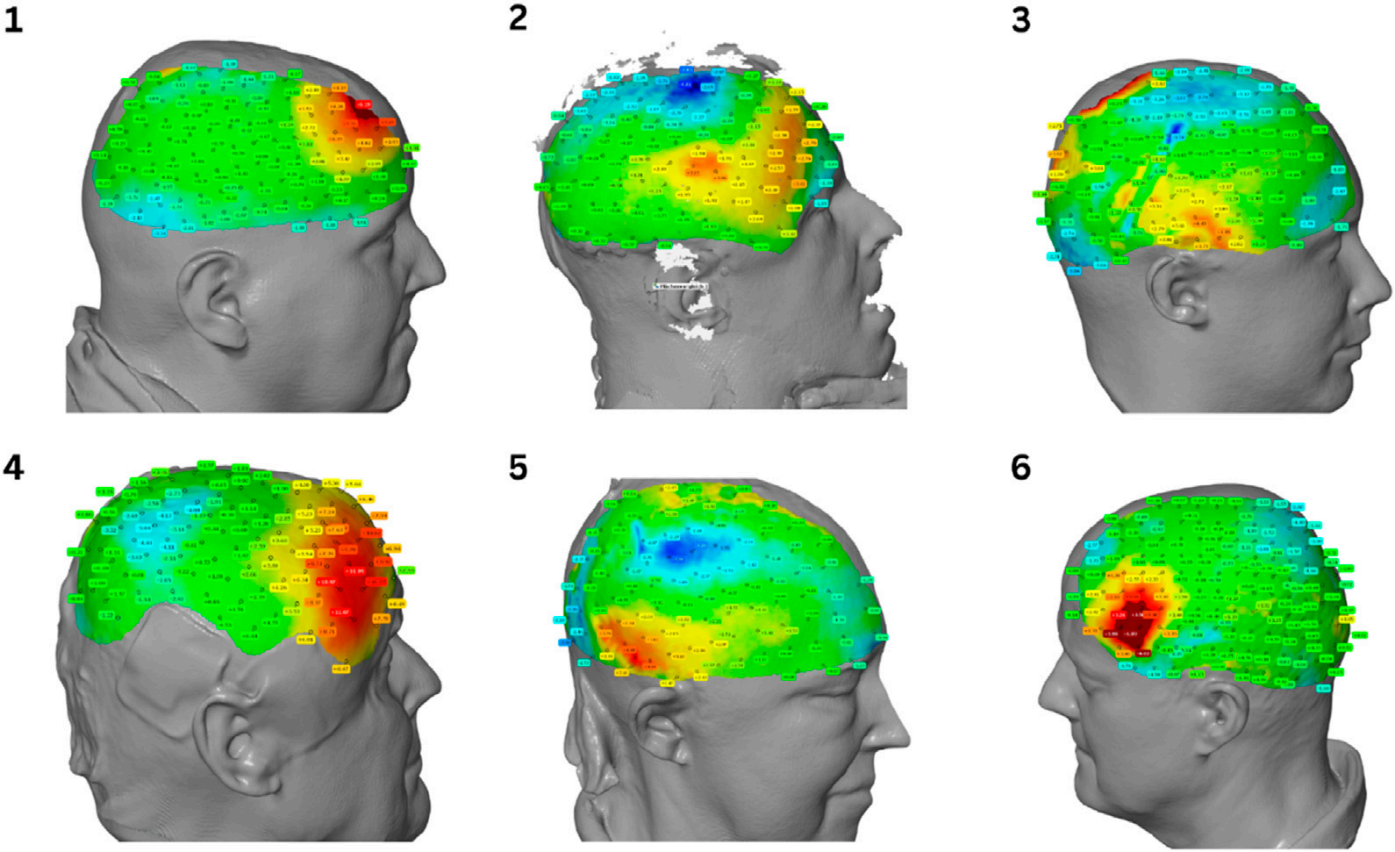
Heatmap comparison of spatial displacement. The color gradient represents the gradient of displacement.

The comparison and overlay of the scan data with the CT data are performed using the Zeiss Inspect software. This software is designed for the evaluation of optically measured 3D surface data. A reference model, in our case the CT data, is superimposed with an actual model, the 3D scan. The alignment of both models is achieved through an Iterative Closest Point (ICP) algorithm, which utilizes transformations (translation and rotation) to align two 3D point clouds and identify common points. A surface comparison as shown in Figure 1, using defined measurement points, illustrates the displacement and generates a heatmap.

## Results

We present the obtained scan of 14 consecutive Patients in form of Heat-map analysis.

**FIGURE 2:**
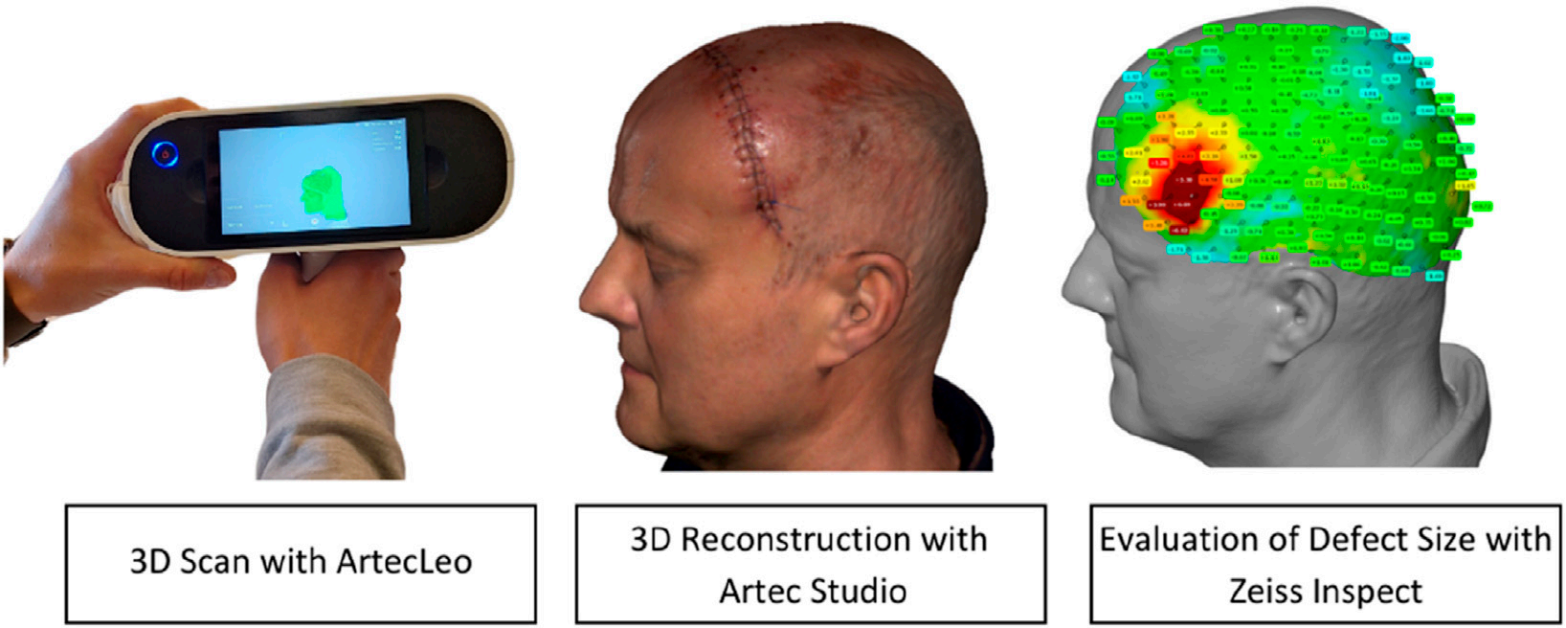
Approach for evaluating defect displacement.

**DIAGRAM 1:**
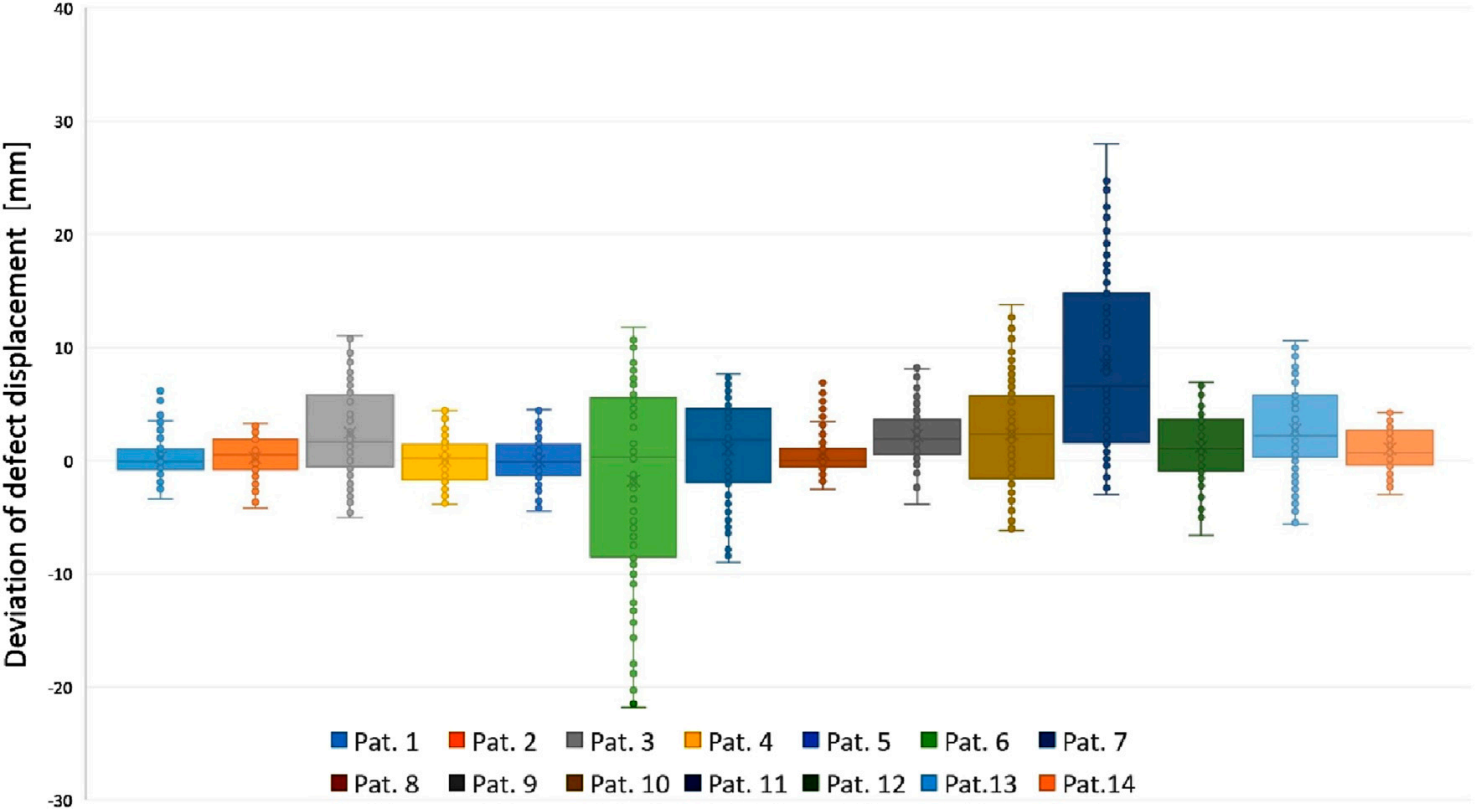
Box plot of defect displacement for the 12 patients.

Figure 2 displays four heatmaps representing patients post craniotomy. Surface comparison reveals a tendency towards shifting of cranial mass. CT scans were acquired in a supine position, whereas the 3D scan was obtained in a seated position. Red areas indicate an increase in mass, while blue to green areas signify a decrease. The deviations range from +28.01 mm to −21.80 mm as shown in Table 1. As shown in Diagram 1, the analysis of the data reveals large differences in the dispersion of the measurements between individual patients.

**TABLE 1:** Deviations defect shift in mm.

Patient	Pat. 1	Pat. 2	Pat. 3	Pat. 4	Pat. 5	Pat. 6	Pat. 7	Pat. 8	Pat. 9	Pat. 10	Pat. 11	Pat. 12	Pat. 13	Pat. 14	
Mean Deviation (mm)	0.33	0.28	2.46	0.05	0.00	−1.79	01.02	0.40	1.89	2.45	8.11	1.23	4.47	0.99	mm
Max. Deviation (mm)	6.19	3.27	11.07	4.45	4.48	11.78	7.68	6.89	8.21	13.78	28.01	6.90	11.82	4.27	mm
Min. Deviation (mm)	−3.38	−4.21	−5.04	−3.86	−4.49	−21.80	−8.98	−2.57	−3.87	−6.16	−3.01	−6.62	−5.60	−2.97	mm

The analysis of the data shows large differences in the dispersion of the measurements between individual patients. These differences suggest that the variability of the measurements can vary considerably from patient to patient. A particularly noteworthy feature of the data is the outliers, whose extent and frequency vary greatly and appear to be individually determined. The central tendency of the data, represented by the median values, shows predominantly positive values. This suggests a general tendency for scalp swelling among the patients.

### Helmet production

The captured point clouds are converted into a closed mesh using ArtecStudio software (Artec3D). This mesh serves as the foundation for the subsequent helmet construction. The helmet design is carried out using Fusion 360 (Autodesk Inc.), utilizing a custom-designed construction template with a defined process that accounts for skin distance, helmet contours, as well as holes and padding. The final perforations are created using nTop (nTopology Inc.). The complete helmet design is individually tailored to each patient.

**FIGURE 3:**
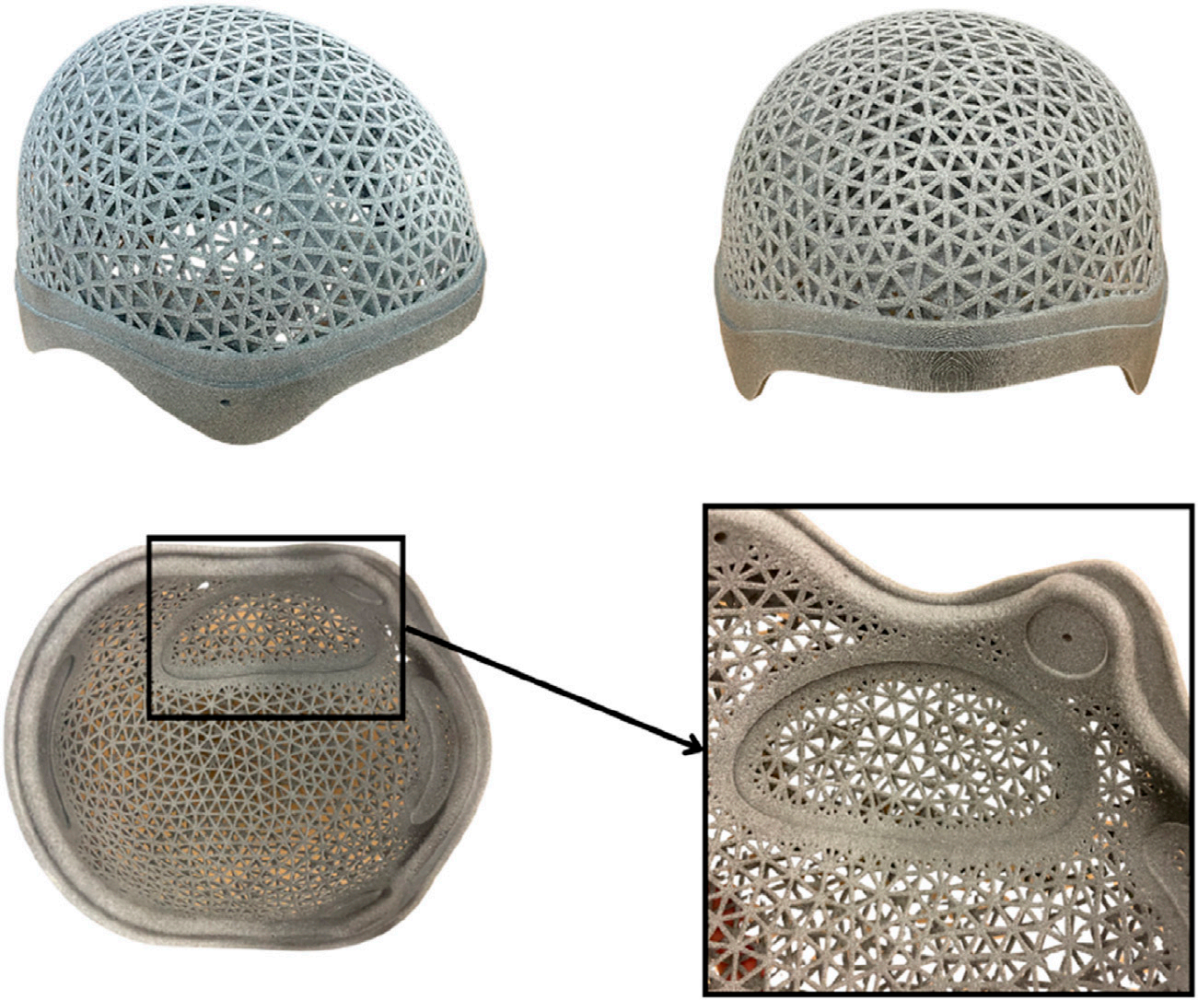
3D printed helmet based on data from an Artec 3D scan.

For helmet production, the 3D scan serves as the basis. The helmet is produced using HP MJF with PA12 as shown in Figure 3. PA12 is an excellent material for protective helmets designed for craniotomized patients due to its biocompatibility, sterilizability, high heat resistance, and durability against moisture, chemicals, and UV radiation, ensuring long-term reliability. Its high strength and toughness provide effective protection against secondary injuries, while the carefully designed structure, featuring internal padding and smooth edges, enhances both comfort and safety. After printing and necessary post-processing, orthopedic technicians finalize the helmet, attaching the padding and chin strap. The helmet is then handed over to the specialist, engineer, and orthopedic technician for final inspection.

## Discussion

We performed a non-invasive data assessment using ArtecLeo and subsequently constructed a 3D printed helm.

The clinical use of this scanning method allowed us to offer a rapid and patient-tailored solution without the necessity of additional CT scan to assess the spatial configuration of the defect, which allows direct mobilization of the patients and provides protection against *de novo* injuries.

The helm does not only protect the patient from the direct impact. Moreover, it is reported that nearly 60% of the patients sleep on the craniectomy site, which might cause preventable micro-damage on daily basis (Pandit et al., 2022). Furthermore, it might even facilitate the inter-hospital mobilization, as the para-medical staff reported on day to day difficulties which patients with proper head-protection do not face (Pandit et al., 2022).

Helmet fabrication using CT imaging is feasible but often yields suboptimal segmentation results for soft tissues. The standard resolution of 1 mm in CT scans is inferior to the higher measurement accuracy provided by 3D scanning. Furthermore, aside from the one-time acquisition cost, the use of a 3D scanner incurs no significant additional expenses.

3D printed helms are making its way to day-to-day clinical practice since 1980 (Rahimy et al., 2021). Beside it´s protective role, the helm may also support the social re-integration of the patient and support the psychosocial wellbeing by covering the visible and oft cosmetically disturbing defect (Colasante et al., 2018).

It is crucial to ensure that the patient’s hair does not distort the scan. Sometimes, hair is not fully captured by the scanners, or if it is, it does not accurately represent the actual head shape. This offset must be eliminated using aids such as a nylon stocking or a Stülpa-Fix bandage. These are carefully placed on the head and conform to its shape.

The scanning process only takes a few moments, with the patient instructed to remain still. For cooperative patients, simply maintaining an upright position suffices. However, if the patient struggles to stay still, medical personnel may be required to assist with immobilization.

Once the scan is complete, the 3D point cloud can be post-processed on a PC. Using ArtecStudio software, the loose 3D points are converted into a watertight 3D model, meaning a closed mesh body with no gaps or holes. This model is then exported as an. STL file for further use in CAD software. In Fusion 360, a predefined design is customized to fit the scan. The inner contour, serving as the head interface, is designed to be breathable, achieved by creating a mesh using nTop. Cutouts are made for padding (on the sides, front, back, and at the defect site).

As described in previous studies (Kropla et al., 2023a; Kropla et al., 2023b) the 3D scan is ideally suited for clinical practice as it can be used without extensive prior knowledge. The 3D scan provides sufficiently accurate, radiation-free 3D models of anatomical structures, benefiting every medical specialty.

The non-invasive form of data assessment seems to be reliable for the helm creation. Furthermore, easy-to-use design allows rapid helm construction without necessity of additional CT-scan.

The helmet only poses a potential hazard or safety risk in cases of misuse. The material has been tested by the manufacturer and is provided to the patient by certified orthopedic technicians. There are no ethical concerns, as everything is done with the patient’s understanding and consent.

## Conclusion

The 3D scan is suitable for surface scans as it provides precise and detailed representations of surface geometries. Thanks to its high precision, it captures even the finest details, is fast, contactless, and versatile for various surface materials and object sizes. However, hair can act as a disturbance factor and should be removed, or a flexible net stocking can be used to ensure accurate capture. Therefore, it is important to perform the scan postoperatively as soon as possible due to hair growth.

Due to radiation exposure, another CT scan is not advisable, while MRI is costly and time-consuming.

Alignment of the CT scan with the 3D scan can be quickly achieved using suitable software, utilizing the ICP (Iterative Closest Point) algorithm. ICP is a computational technique that iteratively minimizes the distance between corresponding points on the two 3D models, aligning them by adjusting their positions and orientations. Anatomical landmarks such as the face or ears serve as clear guides. Generating a heatmap effectively illustrates the displacement of the defect and can be used for comparison purposes, provided the same scaling is applied.

All 14 patients expressed great interest in the new and innovative helmet design, particularly appreciating the ventilation holes, as excessive sweating had always been a significant issue. The precise 3D scanning ensured an optimal fit for each individual helmet.

## Data Availability

The original contributions presented in the study are included in the article/supplementary material, further inquiries can be directed to the corresponding author.
